# Differential Effects of Collagen Prolyl 3-Hydroxylation on Skeletal Tissues

**DOI:** 10.1371/journal.pgen.1004121

**Published:** 2014-01-23

**Authors:** Erica P. Homan, Caressa Lietman, Ingo Grafe, Jennifer Lennington, Roy Morello, Dobrawa Napierala, Ming-Ming Jiang, Elda M. Munivez, Brian Dawson, Terry K. Bertin, Yuqing Chen, Rhonald Lua, Olivier Lichtarge, John Hicks, Mary Ann Weis, David Eyre, Brendan H. L. Lee

**Affiliations:** 1 Department of Molecular and Human Genetics, Baylor College of Medicine, Houston, Texas, United States of America; 2 Department of Physiology and Biophysics, University of Arkansas for Medical Sciences, Little Rock, Arkansas, United States of America; 3 Department of Oral and Maxillofacial Surgery, School of Dentistry, University of Alabama at Birmingham, Birmingham, Alabama, United States of America; 4 Howard Hughes Medical Institute, Baylor College of Medicine, Houston, Texas, United States of America; 5 Department of Pathology, Texas Children's Hospital, Baylor College of Medicine, Houston, Texas, United States of America; 6 Department of Orthopaedics and Sports Medicine, University of Washington, Seattle, Washington, United States of America; Mount Sinai School of Medicine, United States of America

## Abstract

Mutations in the genes encoding cartilage associated protein (*CRTAP*) and prolyl 3-hydroxylase 1 (P3H1 encoded by *LEPRE1*) were the first identified causes of recessive Osteogenesis Imperfecta (OI). These proteins, together with cyclophilin B (encoded by *PPIB*), form a complex that 3-hydroxylates a single proline residue on the α1(I) chain (Pro986) and has cis/trans isomerase (PPIase) activity essential for proper collagen folding. Recent data suggest that prolyl 3-hydroxylation of Pro986 is not required for the structural stability of collagen; however, the absence of this post-translational modification may disrupt protein-protein interactions integral for proper collagen folding and lead to collagen over-modification. P3H1 and CRTAP stabilize each other and absence of one results in degradation of the other. Hence, hypomorphic or loss of function mutations of either gene cause loss of the whole complex and its associated functions. The relative contribution of losing this complex's 3-hydroxylation versus PPIase and collagen chaperone activities to the phenotype of recessive OI is unknown. To distinguish between these functions, we generated knock-in mice carrying a single amino acid substitution in the catalytic site of P3h1 (*Lepre1^H662A^*). This substitution abolished P3h1 activity but retained ability to form a complex with Crtap and thus the collagen chaperone function. Knock-in mice showed absence of prolyl 3-hydroxylation at Pro986 of the α1(I) and α1(II) collagen chains but no significant over-modification at other collagen residues. They were normal in appearance, had no growth defects and normal cartilage growth plate histology but showed decreased trabecular bone mass. This new mouse model recapitulates elements of the bone phenotype of OI but not the cartilage and growth phenotypes caused by loss of the prolyl 3-hydroxylation complex. Our observations suggest differential tissue consequences due to selective inactivation of P3H1 hydroxylase activity versus complete ablation of the prolyl 3-hydroxylation complex.

## Author Summary

The prolyl 3-hydroxylase complex serves to hydroxylate a single residue in type I collagen and also serves as a collagen chaperone. The complex is comprised of prolyl 3-hydroxylase 1, cartilage associated protein, and cyclophilin B. Mutations have been identified in the genes encoding the complex members in patients with recessive Osteogenesis Imperfecta. Recent data suggest that prolyl 3-hydroxylation of collagen does not alter the stability of collagen but may rather mediate protein-protein interactions. Additionally, the collagen chaperoning function of the complex is an important rate limiting step in the modification of type I collagen. Irrespective of whether patients with mutations in the genes encoding the members of the prolyl 3-hydroxylase complex have hypomorphic or complete loss of function alleles, either circumstance leads to the loss of both functions of the prolyl 3-hydroxylase complex. Thus, it is unknown how collagen chaperoning and/or hydroxylation affect bone and cartilage homeostasis. In this study, we generated a mouse model lacking the prolyl 3-hydroxylation activity of the complex while maintaining the chaperoning function. We found that the hydroxylase mutant mice have normal cartilage and normal cortical bone but decreased trabecular bone, suggesting that there is a differential requirement for hydroxylation in different tissues.

## Introduction

Although dominant mutations in the type I procollagen genes, *COL1A1 and COL1A2*, account for the majority of patients with Osteogenesis Imperfecta (OI) (#166200, #166210, #166220, #259420, #259440, #610682, #610915, #610967, #610968, #613848, #613849, #613982, #614856, #615066), the disorder can also be inherited in an autosomal recessive manner [1]. A mutation in cartilage associated protein (*CRTAP*) (*605497) was first identified by Morello et. al. in a class of patients with recessive OI [2]. CRTAP functions in a complex with prolyl 3-hydroxylase 1 (P3H1) (*610339) and cyclophilin B (CYPB) (*123841) to 3-hydroxylate a unique proline, Pro986, of the α1(I) chain and also to chaperone collagen trimer assembly [3]–[5]. Additionally, other clade A fibrillar collagens, such as collagen type II, are similarly hydroxylated. Subsequently, mutations in both leucine and proline enriched proteoglycan (*LEPRE1*), encoding P3H1, and peptidylprolyl isomerase b (*PPIB*), encoding CYPB, were identified in other patients with recessive forms of OI [6]–[13]. Mutations in additional genes have since been identified in recessive OI, revealing novel mechanisms of disease through alterations in post-translational collagen modification, trafficking and signaling.

Knockout mice have been created for each of the three genes that encode the prolyl 3-hydroxylase complex and these mice recapitulate the phenotype observed in recessive OI patients. *Crtap^−/−^ and Lepre1^−/−^* mice display osteochondrodysplasia, severe low bone mass, kyphosis, rhizomelia and collagen fibrils with irregular diameter [2], [14]. Although the *Ppib^−/−^* mice do not have rhizomelia, they also have a low bone mass phenotype with kyphosis and wider collagen fibrils [15]. Together, these findings highlighted the importance of this complex in collagen modification and maintenance of bone mass.

Since null mutations in either *LEPRE1* or *CRTAP* can result in recessive forms of Osteogenesis Imperfecta (OI) with almost identical features, P3H1 and CRTAP were hypothesized to stabilize each other [16]. Indeed, western blot and immunofluorescence studies demonstrated absence of both CRTAP and P3H1 in fibroblasts isolated from patients carrying either *LEPRE1* or *CRTAP* mutations alone [16], [17]. These data supported the notion that CRTAP and P3H1 proteins stabilize each other in the endoplasmic reticulum.

The present study aimed to determine the relative contribution of the hydroxylation activity of the P3H1 complex to the pathogenesis of recessive OI. This important question was addressed *in vitro* and *in vivo* by introducing a point mutation in the catalytic domain of P3H1 that inactivated hydroxylase activity while preserving the protein secondary structure and the CRTAP/P3H1/CypB complex integrity.

## Results

### Determining amino acids important for the hydroxylase function of P3H1

**Figure 1:**
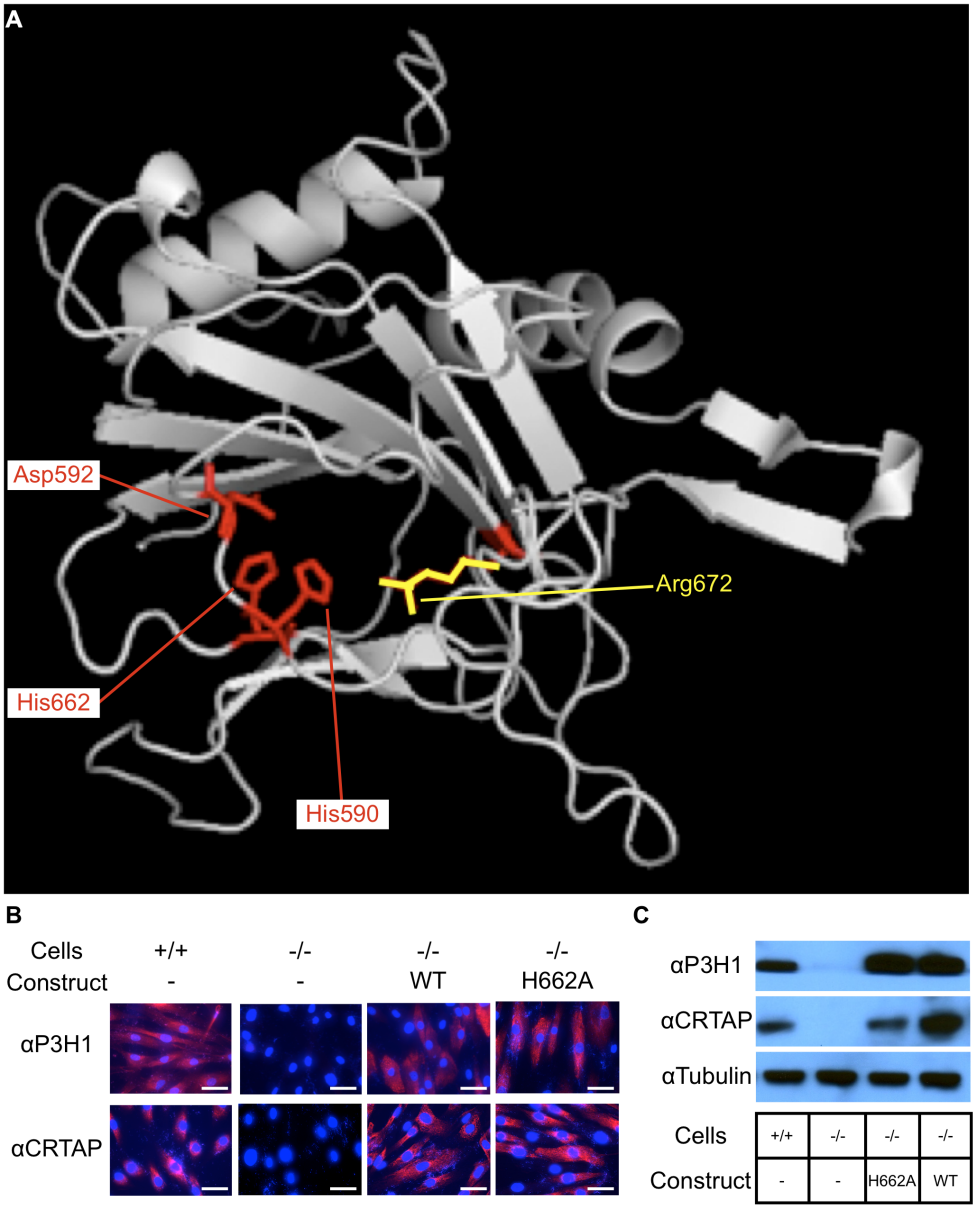
Residues important for hydroxylase function and catalytic mutations rescue the stability of CRTAP. Evolutionary trace analysis identified residues important for the function of P3H1 by comparing each residue within the dioxygenase domain family. The highest ranking residues are mapped onto the dioxygenase domain responsible for prolyl 3-hydroxylation (A). These residues include 3 that interact with iron (His590, Asp592, and His662) shown in red and 1 that interacts with 2-oxoglutarate (Arg672) shown in yellow. An alanine substitution was introduced at HIS662 (H662A) to deactivate the hydroxylase function. To test whether the mutant P3H1 was able to rescue the stability of CRTAP, we transduced immortalized *LEPRE1* loss of function fibroblasts with WT or H662A mutant *LEPRE1* cDNA and assayed for the presence of CRTAP by immunofluorescence and immunoblot. Here we demonstrate that the stability of CRTAP is rescued by immunofluorescence (B) and by immunoblot (C).

To accomplish our goal of inactivating the hydroxylase function of P3H1, we employed an evolutionary trace algorithm to identify conserved residues that are essential for its enzymatic function. The four top ranking residues identified were the catalytic triad residues (HIS590, HIS662, and ASP592) and the 2-oxoglutarate binding residue (ARG672) (**Figure 1A**). The importance of these residues for hydroxylase activity was confirmed by earlier literature [18], [19]. Conversion of the catalytic triad histidines or aspartic acid to either alanine or glutamate abolished the enzymatic activity of Prolyl 4-hydroxylase activity in *A. thaliana* [20]. Similarly, conversion of the 2-oxoglutarate binding arginine to alanine also abolished the hydroxylase activity, suggesting that substituting the corresponding sites in P3H1 with an alanine could potentially inactivate its hydroxylase function [20]. Since alanine is a non-bulky, uncharged amino acid that can mimic the secondary structure of many other amino acids, we opted for this substitution rather than a glutamate substitution to preserve the structural integrity of P3H1.

### Rescue of CRTAP stability by mutant P3H1 in cell culture

Since the stability of the prolyl 3-hydroxylase complex is dependent on the interaction of P3H1 and CRTAP, it was important to verify that the mutation introduced into *LEPRE1* did not disrupt its ability to form a stable complex with CRTAP and CYPB. To do this we first used an *in vitro* approach. We used immortalized patient fibroblasts carrying a loss of function mutation in *LEPRE1* and tested whether the expression of 4 different *LEPRE1* constructs containing alanine substitutions at H590, D592, H662, or R672 were able to restore the stability of CRTAP by immunofluorescence and immunoblot assays. We found that the mutant expression construct converting H662 to alanine (P3H1^H662A^) was the most effective at rescuing CRTAP compared with the others or the un-transduced *LEPRE1* loss of function cells (**figure 1B, C**). These findings are consistent with P3H1^H662A^ being able to interact with CRTAP and to restore it to the ER. Although P3H1^H662A^ was not assayed for enzymatic activity, mutating the corresponding residue to an alanine in Prolyl 4-hydroxylase in *A. thaliana* resulted in complete inactivation of the hydroxylase activity, suggesting the P3H1^H662A^ mutant is likely to be inactive [20].

### Demonstration that *Lepre1^H662A/H662A^* knock-in mice lack Pro986 collagen hydroxylation

**Figure 2:**
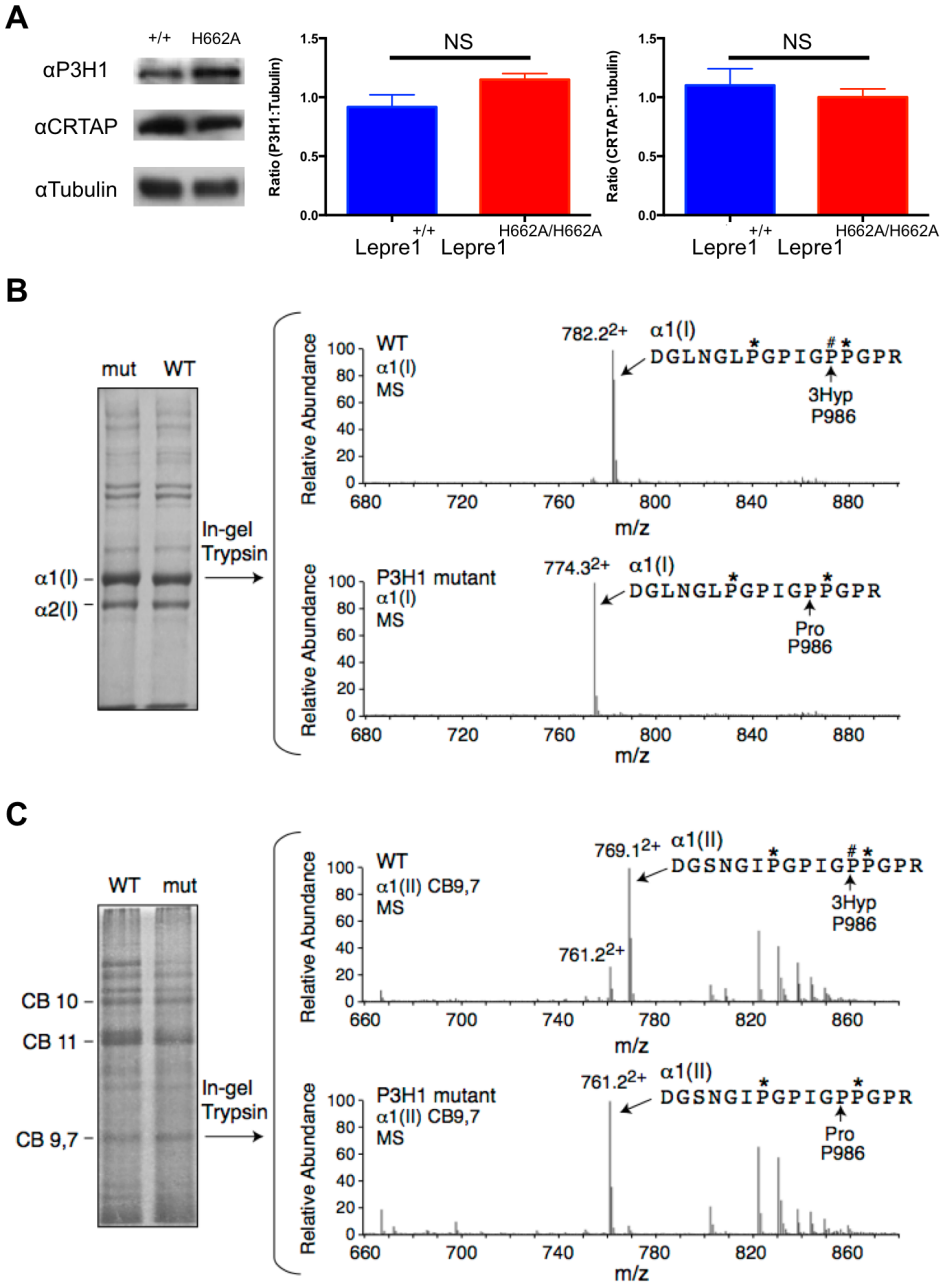
Loss of Prolyl 3-hydroxylation at Pro986 in type I collagen in bone and type II collagen in cartilage. Upon generation of the *Lepre1^H662A/H662A^* mice, we confirmed the stability of both P3H1 and CRTAP by western blot using protein isolated from mouse calvaria (A, experiments repeated 3 times). Comparing protein isolated from the *Lepre1^H662A/H662A^* and the *Lepre1^+/+^* mice, we found no differences in the levels of P3H1 and CRTAP when compared to γ-Tubulin. Analysis of prolyl 3-hydroxylation of Pro986 on the α(1) chain of type I collagen in bone using mass spectrometry demonstrates complete loss of 3-hydroxylation in the *Lepre1^H662A/H662A^* mice when compared to *Lepre1^+/+^* littermates (B). Similarly, analysis of the Pro986 site on the α1 chain of type II collagen in cartilage demonstrates a reduction to 9% 3-hydroxylation in the *Lepre1^H662A/H662A^* mice and is similar to what was reported in *Crtap^−/−^* mice (C).

**Figure 3:**
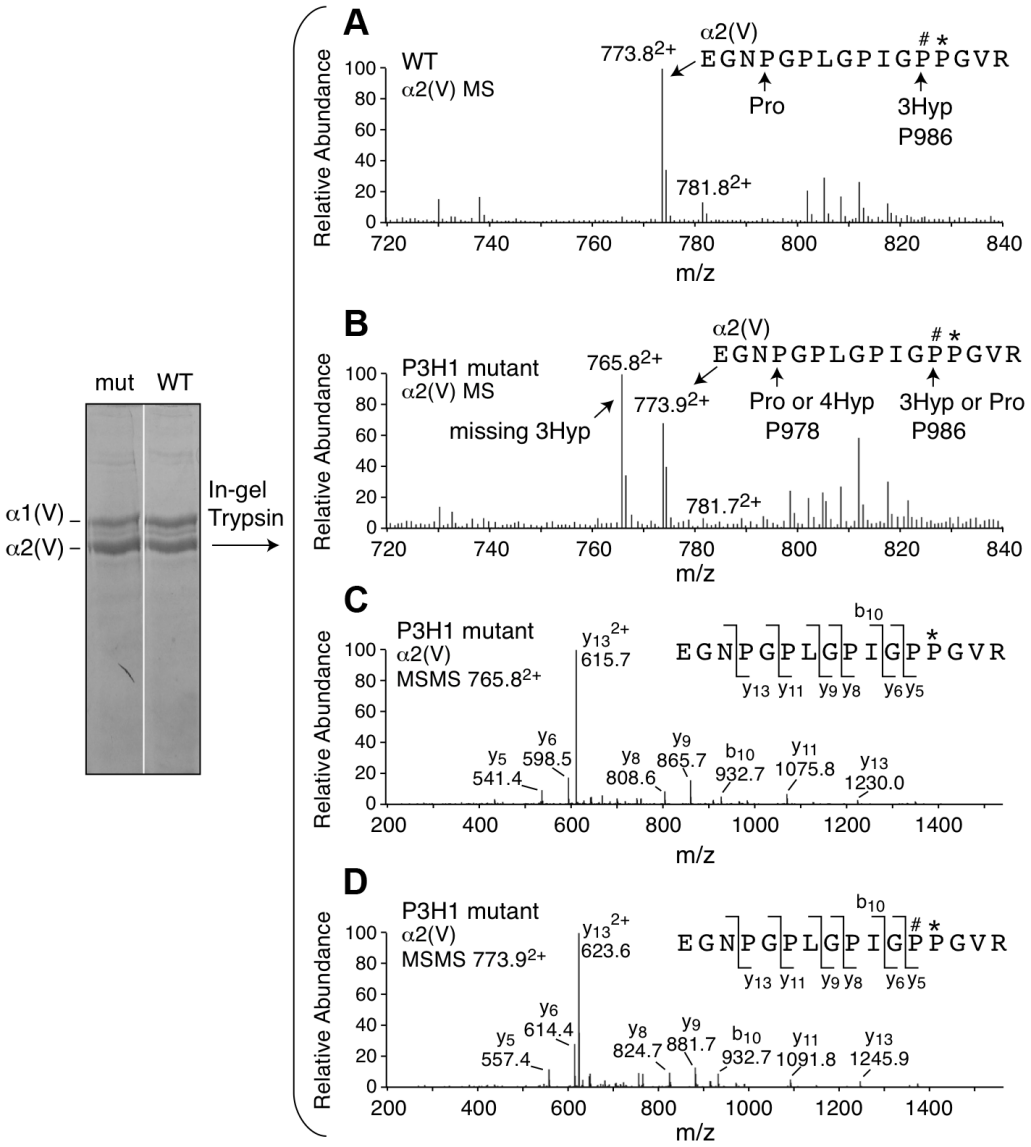
Prolyl 3-hydroxylation at Pro986 in the α2(V) collagen chain. Mass spectral analysis of Pro986 hydroxylation in tryptic peptides from the α2(V) chain of bone from *Lepre1^+/+^* and *Lepre1^H662A/H662A^* mice (A and B respectively) shows a marked reduction in hydroxylation at this site. The MS/MS fragmentation patterns shown in C and D identified the 765.8^2+^ peptide and its 3-hydroxylated version 773.9^2+^. A portion (40%) of the latter ion was also found by MS/MS to be contributed by a version lacking 3-Hyp but containing 4-Hyp at P978 (taken into account in the 3-Hyp quantitation).

We generated knock-in mice carrying the H662A mutation at the *Lepre1* locus (*Lepre1^H662A/H662A^*) (**figure S1**). Importantly, we verified that CRTAP is also restored *in vivo* in the *Lepre1^H662A/H662A^* mice compared with wild-type littermates by confirming its presence by western blot using protein isolated from P1 calvaria (**figure 2A**). Since the Pro986 residue of α1(I), α1(II) and α2(V) procollagen chains is normally fully hydroxylated and complete loss of the P3H1 complex abolishes hydroxylation at these sites [2], [14], [15], [17], we analyzed the hydroxylation status of these residues in the *Lepre1^H662A/H662A^* mice to assess the *in vivo* enzymatic activity of P3H1^H662A^. Tandem mass spectrometry showed loss of prolyl 3-hydroxylation (3-Hyp) at Pro986 in the α1(I) chain and a residual 21% 3-Hyp in the α2(V) chain from bone (**figure 2B** **,** **3**). Pro978 of the bone α2(V) chain remained minimally 4-hydroxylated, similar to the level observed in wild-type littermate bone (**figure 3**). This is in contrast to *Crtap^−/−^* mice in which 3-Hyp at Pro986 was missing but in effect replaced by 4-Hyp at Pro978 apparently as a consequence of over-modification in comparison to wild-type mice [17]. In cartilage, tandem mass spectrometry showed specifically a residual 9% 3-Hyp at Pro986 in the α1(II) chain of the *Lepre1^H662A/H662A^* mice (**figure 2C**), which is comparable to the 6% 3-Hyp observed in *Crtap^−/−^* mice (not shown). These findings are summarized in **Table 1** together with the hydroxylation status of other partially occupied 3-Hyp sites in bone and cartilage collagens. The loss of 3-Hyp appears to be specific to Pro986 of types I, II and V collagens similar to findings in mouse models with complete loss of the P3H1 complex [2], [14], [15], [17].

**Table 1:** Hydroxylation status at different collagen sites.

Tissue	Collagen	Position	*Lepre1^+/+^* (SD)	*Lepre1^H662A/H662A^* (SD)
Bone	α1(I)	P986	93 (4)	3 (1.8)
Bone	α2(I)	P707	21 (11)	16 (6)
Bone	α2(V)	P986	97 (1)	21 (6)
Bone	α2(V)	P944	63 (6)	66 (9)
Bone	α2(V)	P707	54 (11)	41 (9)
Cartilage	α1(II)	P986	87 (9)	9 (3)
Cartilage	α1(II)	P944	70 (21)	74 (9)

Table shows the percent 3-hydroxylation of proline at known sites of potential occupancy in type I and type II collagen of bone and cartilage determined by mass spectrometry.

### Effects on lysine hydroxylation and cross-linking

Collagen cross-linking in bone was studied by determining the ratio of hydroxylysyl pyridinoline to lysyl pyridinoline (HP/LP). This ratio reflects the hydroxylation status of those triple-helical lysines at K87 and K930 in α1(I) and/or K87 and K933 in α2(I) that had participated in cross-link formation. In the *Lepre1^H662A/H662A^* mice, there was an increase in the HP/LP ratio compared to wild-type littermates indicating that there may have been an overall increase in hydroxylation at one or both of these sites. However, mass spectral analysis of linear (uncross-linked) sequences from the same helical cross-linking sites prepared by digestion either with bacterial collagenase or trypsin showed no significant differences between *Lepre1^H662A/H662A^* and wild-type bone collagen. Residue α1(I) K930 was 98% hydroxylated and non glycosylated in both genotypes and α1(I)K87 was 92% hydroxylated in wild-type and 93% in *Lepre1^H662A/H662A^*. So the difference in HP/LP ratio may be due to altered hydroxylation at the homologous sites in the α2(I) chain but we did not acquire informative peptides from the latter. Informative peptides from non cross-linking lysine sites also showed no significant differences between wild-type and *Lepre1^H662A/H662A^* bone. For example, α1(1) K174 was essentially all galactosyl Hyl in both genotypes and α2(1) K219 was 70% hydroxylated in wild-type and 77% in *Lepre1^H662A/H662A^*. Thus, no evidence of generalized over-modification was found from these site-specific mass spectral results.

The content of HP+LP in the bone collagen was not significantly altered (**Table 2**). Consistent with the latter observation, *Lepre1^H662A/H662A^* mice showed no significant difference in the ratio of telopeptide hydroxylysine to lysine in the extracted bone collagen α1(I) chains when compared to their wild-type littermates based on the mass spectral ratio of Hyl to Lys versions of the telopeptides not involved in cross-linking (**Table 2**). A caution here is that this estimate comes from a relatively minor fraction of the total matrix collagen.

**Table 2:** Pyridinoline content and telopeptide hydroxylation of bone collagen.

	*Lepre1^+/+^*	*Lepre1^H662A/H662A^*	P-value
HP (moles/mole)	0.20 (0.1)	0.17 (0.03)	NS
LP (moles/mole)	0.02 (0.0)	0.01 (0.00)	NS
HP/LP	8.7 (0.5)	14.0 (1.5)	P<0.02
α1(I)N-telo Hyl	96%	95%	
α1(I)C-telo Hyl	72%	76%	

Hydroxylysylpyridinoline (HP) and lysylpyridinoline (LP), were measured in bone collagen. Listed are the HP and LP contents (moles/mole of collagen), and the molar ratio (HP/LP) for *Lepre1^H662A/H662A^* and *Lepre1^+/+^* mice showing a significant increase in the HP/LP ratio in the *Lepre1^H662A/H662A^* mice consistent with more hydroxylated lysines at helical cross-linking sites (N = 5, p<0.02). The percentage hydroxylation of α1(I) chain N- and C- telopeptides from mass spectrometry appeared not to be affected (N = 1, both genotypes, p = NS).

### Skeletal phenotype of *Lepre1^H662A/H662A^* mice

**Figure 4:**
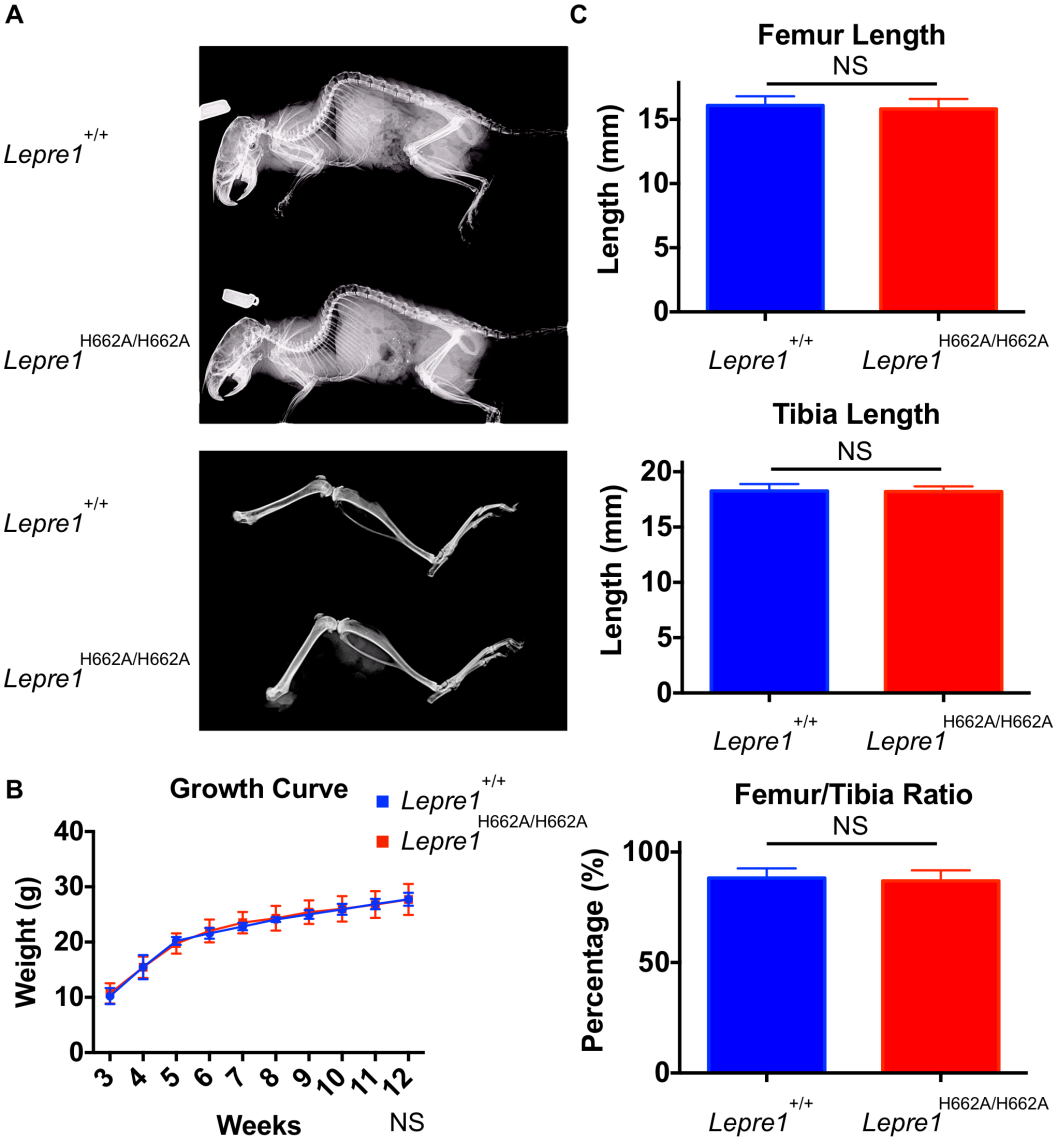
*Lepre1^H662A/H662A^* mice are normal in gross morphology. Analysis at 3 months by X-ray shows no difference in terms of skeletal patterning between genotypes (A). By growth curve analysis, there is no difference in weight at any time point over 3 months between *Lepre1^+/+^* and *Lepre1^H662A/H662A^* mice (B). We assessed rhizomelia by measuring the length of both the femur and tibia and computing the ratio. Comparing *Lepre1^+/+^* to *Lepre1^H662A/H662A^* mice, we observed no difference in the length of the femur, tibia, or their ratio (C). N = 10, both genotypes.

At birth, the *Lepre1^H662A/H662A^* mice are indistinguishable from their wild-type littermates by gross physical appearance (data not shown). Radiographs at 3 months and 6 months of age showed normal skeletal patterning and no evidence of skeletal deformity such as kyphoscoliosis (**figure 4A** **, not shown**). Previous studies of the *Crtap^−/−^* and *Lepre1^−/−^* mice showed a disorganization of the growth plate causing smaller body size with shortening of the proximal long bone segments (rhizomelia) [2], [14]. To assess growth, the *Lepre1^H662A/H662A^* mice and their wild-type littermates were weighed weekly until 3 months of age. At all time points, the weight of *Lepre1^H662A/H662A^* mice was not statistically different from that of wild-type littermates (**figure 4B**). Similarly, there were no significant differences between the lengths of the femur, tibia, nor the femur/tibia ratio, thus excluding any rhizomelic defect in the *Lepre1^H662A/H662A^* mice [2], [14] (**figure 4C**).

**Figure 5:**
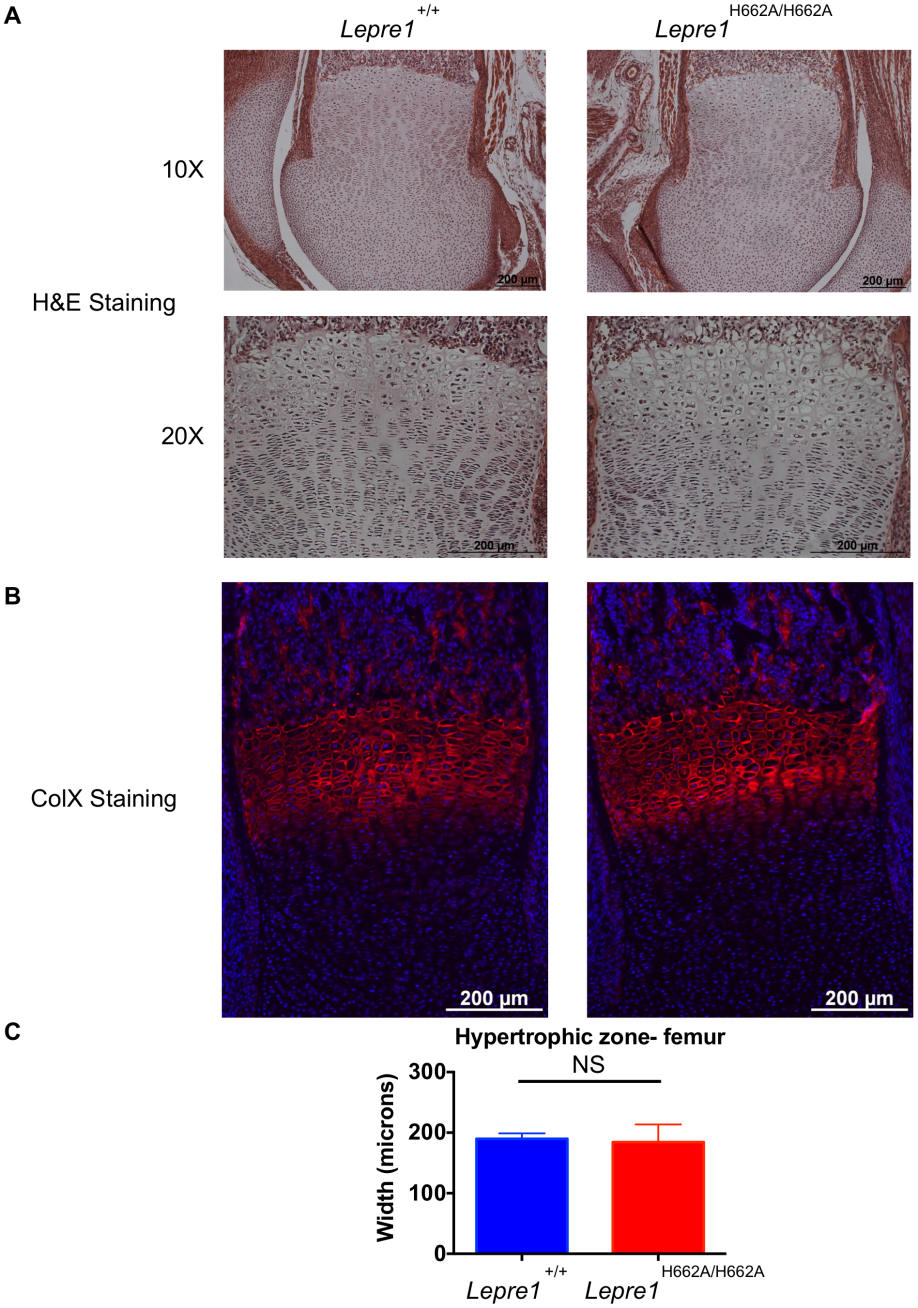
*Lepre1^H662A/H662A^* mice have a normal femoral hypertrophic zone. Since the *Lepre1^−/−^* animals showed disorganization of the hypertrophic zone, we assessed the hypertrophic zone (P1) of *Lepre1^H662A/H662A^* mice by H&E staining (A) and by specifically marking the hypertrophic zone using an antibody directed against type×collagen (B). The hypertrophic zone of the femur of the *Lepre1^H662A/H662A^* mice are indistinguishable from their wild-type littermates (B) and this is confirmed by quantifying the width of the hypertrophic zone in which there is no difference in the width between genotypes (C) (N = 10, both genotypes).

Since the *Lepre1^−/−^* mice have a dysplasia of the growth plate that also affects the hypertrophic chondrocytes, we performed histology and specific staining of the hypertrophic zone with an antibody directed towards type X collagen in *Lepre1^H662A/H662A^* mice at P1 [14]. No defects were observed in the *Lepre1^H662A/H662A^* mice compared to their wild-type littermates (**figure 5A, B** **; figure S2A, B**). Additionally, we observed no difference in the width of the hypertrophic zone between the two genotypes (N = 8, p-value = NS) (**figure 5C** **; figure S2C**). Collectively, the normal growth curve, normal femur to tibia ratio and normal hypertrophic zone suggest that the cartilage in the long bones of *Lepre1^H662A/H662A^* mice is indeed normal despite of loss of Pro986 hydroxylation in type II collagen.

**Figure 6:**
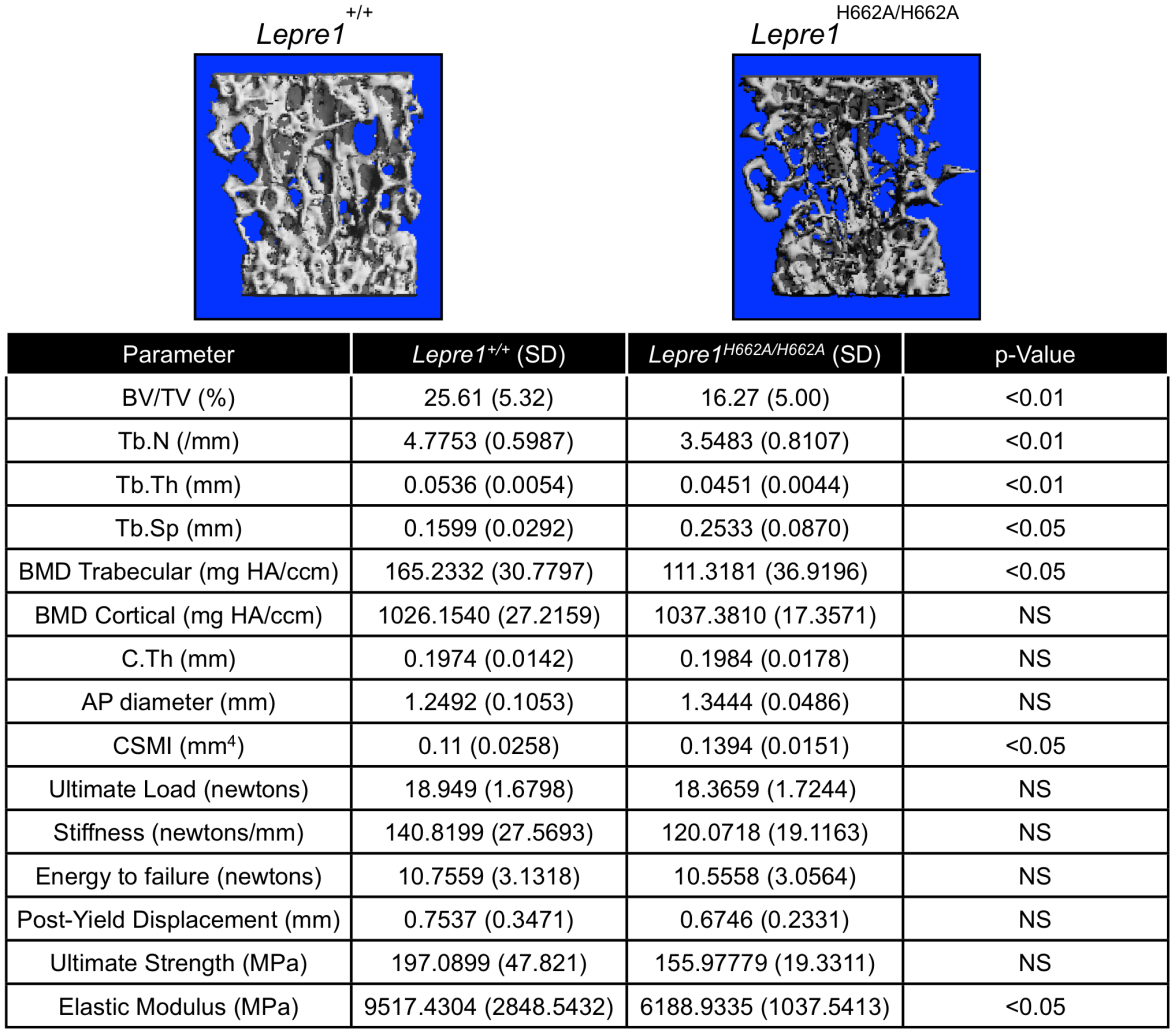
Micro-Computed Tomography and cortical biomechanical analyses. 3D reconstruction of spines from the *Lepre1^H662A/H662A^* mice compared to wild-type littermates. The *Lepre1^H662A/H662A^* mice have less trabecular bone as quantified by reduced bone volume (BV/TV), reduced trabecular number (Tb.N), reduced trabecular thickness (Tb.Th), reduced trabecular bone mineral density (BMD), and increased trabecular separation (Tb.Sp). Cortical parameters are similar to wild-type, as quantified by normal cortical BMD or stiffness and force to failure. These results suggest that the *Lepre1^H662A/H662A^* mice have normal cortical bone but reduced trabecular bone. (N = 10, each genotype).

By assessing the femurs and spines at 3 months of age (n = 10, each genotype) by micro-computed tomography, we found that the *Lepre1^H662A/H662A^* mice had cortical bone mineral density and cortical thickness values comparable to wild-type littermates (**figure 6**). In addition, while the biomechanical analysis of femurs by 3-point bending test (n = 7, each genotype) demonstrated no differences in the extrinsic biomechanical properties (ultimate load, stiffness, energy to failure and post-yield displacement), the geometric value, cross-sectional moment of inertia, was increased and the elastic modulus, an intrinsic material property, was decreased in the *Lepre1^H662A/H662A^* mice compared to wild-type littermates (**figure 6**). Moreover, the *Lepre1^H662A/H662A^* mice have less trabecular bone when compared to wild-type littermates, which is quantified by decreased bone volume over tissue volume (BV/TV), decreased trabecular number (Tb.N), decreased trabecular thickness (Tb.Th), and increased trabecular separation (Tb.Sp) (**figure 6**). These findings are partly in contrast to what was described for *Lepre1^−/−^* mice, which are characterized by a decrease in both trabecular and cortical bone mineral density [14], and cortical stiffness and force to failure of femurs [14].

**Figure 7:**
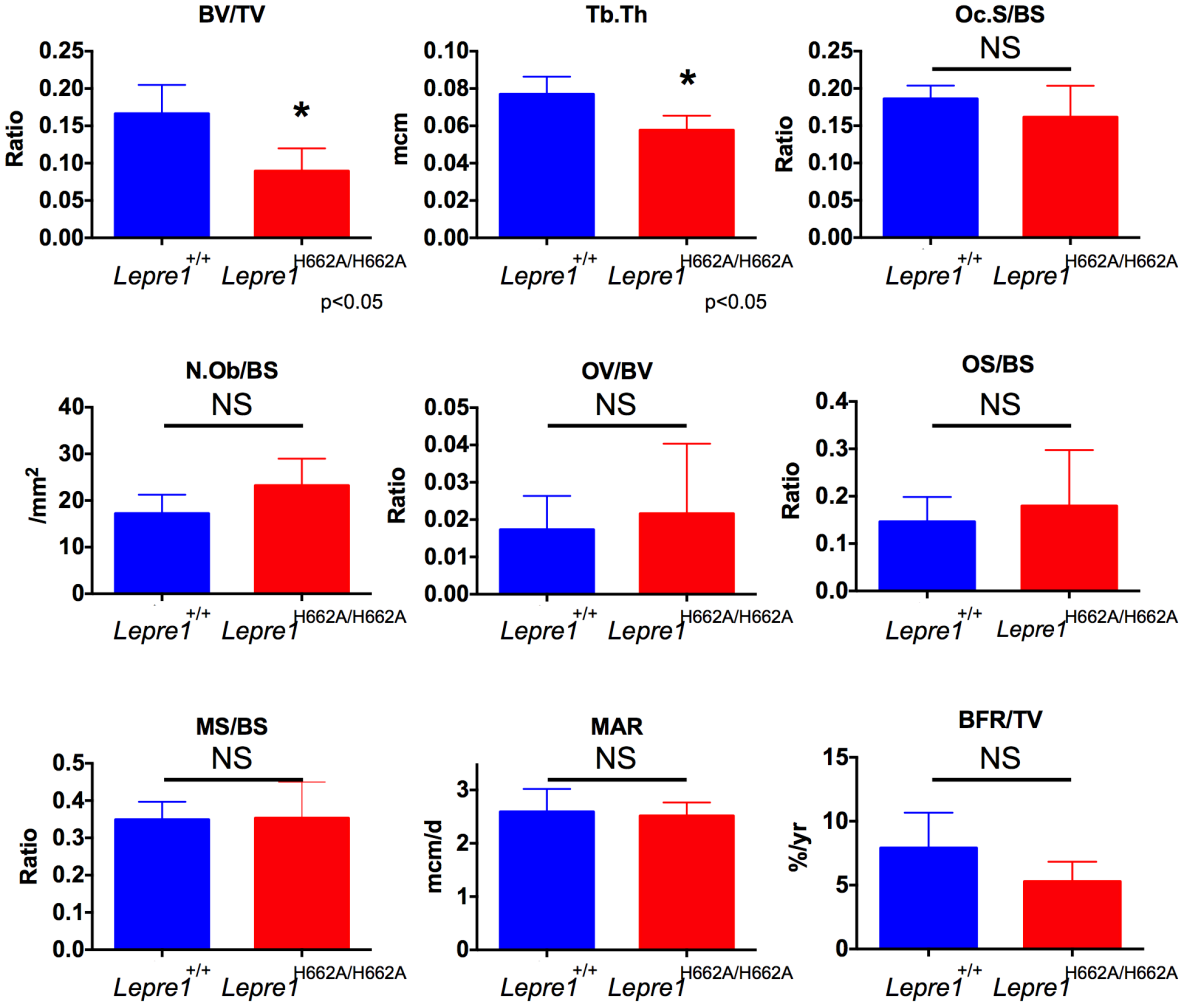
Histomorphometry of *Lepre1^H662A/H662A^* mice. By histomorphometry, we were able to confirm the low trabecular bone mass phenotype in the *Lepre1^H662A/H662A^* mice as quantified by decreased bone volume (BV/TV) and trabecular thickness (Tb.Th). We observed no differences in osteoblast and osteoclast parameters, as measured by the number of osteoblasts (N.Ob/BS) and osteoclast surface (OcS/BS). We observed no difference in the kinetic indices of bone formation, as measured by mineral apposition (MAR), mineralizing surface (MS/BS) and bone formation rate (BFR/TV) and in osteoid parameters, as measured by osteoid volume (OV/BV) and osteoid surface (OS/BS) (mean ± SD, N = 9, both genotypes).

To further study the trabecular bone phenotype in the *Lepre1^H662A/H662A^* mice, bone histomorphometric analysis was conducted on 3-month spines (n = 9). A statistically significant decrease in bone volume over tissue volume and trabecular thickness confirmed the low bone mass phenotype (**figure 7**) [2]. However, no differences in the osteoblast number (N.Ob/BS), osteoclast surface over bone surface (Oc.S/BS), osteoid parameters (osteoid volume over bone volume and osteoid surface over bone surface) or bone formation rate were observed between the two genotypes (**figure 7**).

### Collagen fibril ultrastructure, secretion rate, and steady-state levels

**Figure 8:**
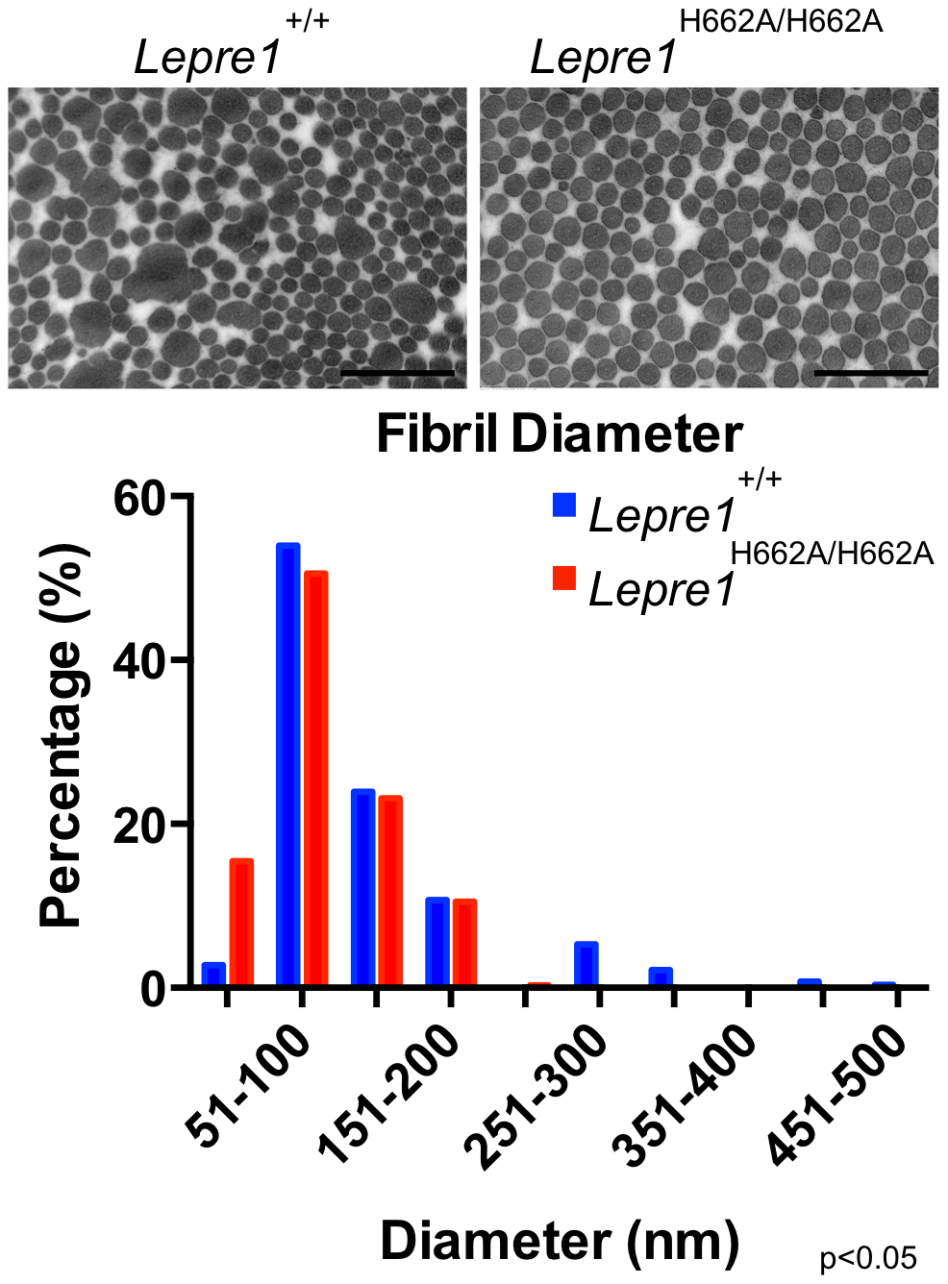
*Lepre1^H662A/H662A^* mice have smaller collagen fibril diameter. Transmission EM analysis of collagen fibrils from skin revealed fibrils more homogenous in size in the *Lepre1^H662A/H662A^* mice as compared to the wild-type littermates. Additionally, the collagen fibril diameters are slightly smaller, as quantified by a slight increase in the proportion of smaller diameter collagen fibrils. (bar = 100 nm). (N = 3,150 collagen diameters measured per animal, p<0.05).

The morphology of collagen fibrils was then analyzed at the ultrastructural level in skin biopsies from *Lepre1^H662A/H662A^* mice. The electron micrographs showed collagen fibrils that were more homogeneous in diameter compared to wild-type controls (**figure 8**). This suggested that collagen trimers may not be efficiently assembled into higher order collagen fibrils as reflected by an increase in the proportion of smaller diameter collagen fibrils in the *Lepre1^H662A/H662A^* skin (N = 3, 150 collagen diameters measured per animal, p<0.05) (**figure 8**).

**Figure 9:**
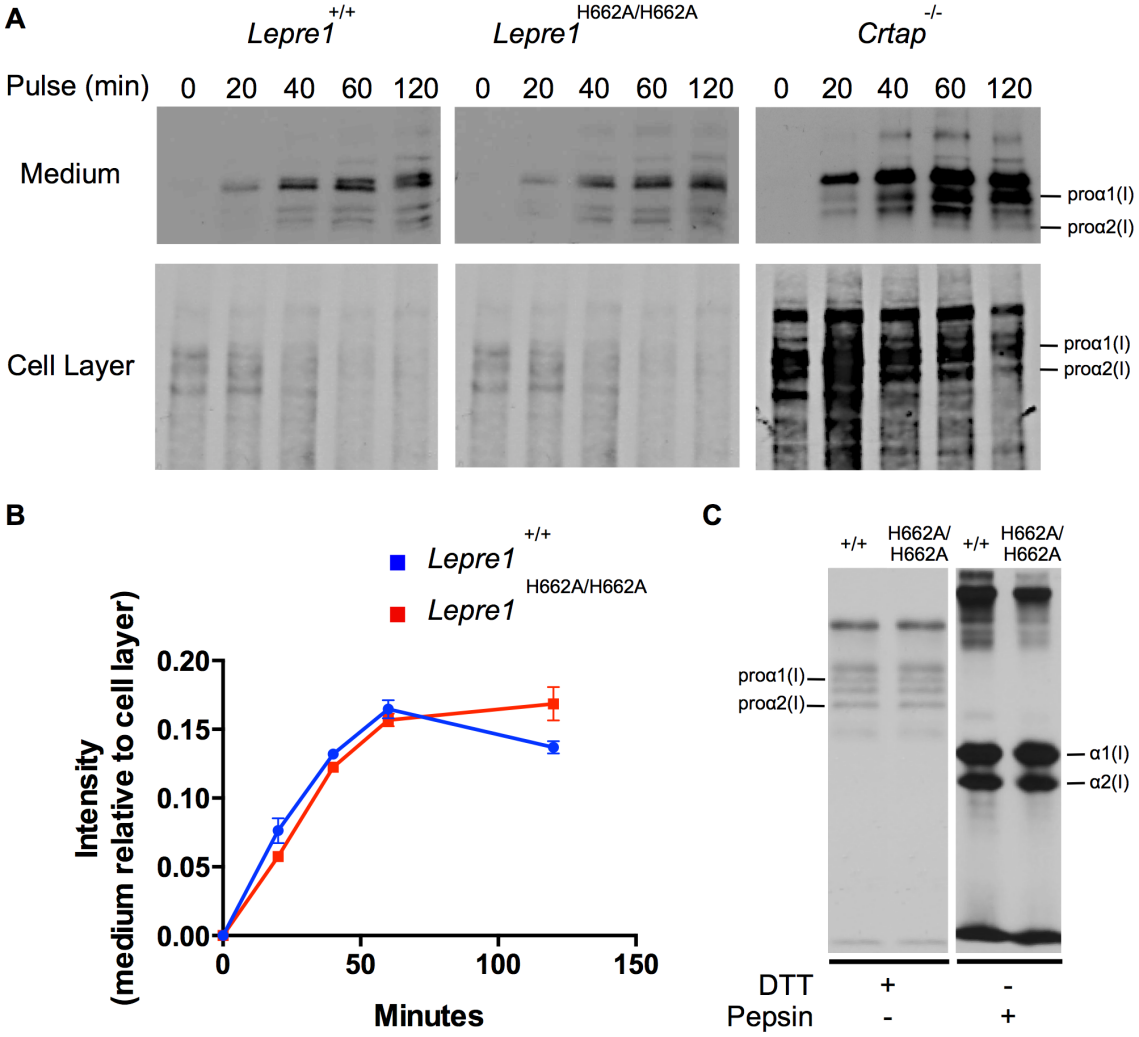
*Lepre1^H662A/H662A^* fibroblast procollagen secretion rate and collagen modification is normal. Analysis of procollagen secretion by the collagen pulse-chase assay suggests that the procollagen secreted from *Lepre1^H662A/H662A^* fibroblasts is similar to *Lepre1^+/+^* fibroblasts (A, B). Additionally, there does not appear to be a decrease in the amount of procollagen secreted from the *Lepre1^H662A/H662A^* fibroblasts in comparison to *Lepre1^+/+^* fibroblasts (A, B). These findings are in contrast to that of the *Crtap^−/−^* fibroblasts, which have an increase in the rate of procollagen secretion (A). Collagen modification was assessed using the collagen steady-state assay. We observed no difference in the migration pattern of procollagen and collagen isolated from *Lepre1^+/+^*(+/+) and *Lepre1^H662A/H662A^* (H662A/H662A) fibroblasts (C). These assays were repeated three times.

We then analyzed the collagen secretion rate, as measured by pulse-chase assays in dermal fibroblasts. The rate and amount of procollagen secreted from the *Lepre1^H662A/H662A^* fibroblasts were apparently similar to those secreted by wild-type fibroblasts (repeated 3 times) (**figure 9A, B**). These findings contrast with the delayed procollagen secretion observed in the *Lepre1^−/−^* mice and are apparently different from the increase in the collagen secretion rate observed in the *Crtap^−/−^* fibroblasts (**figure 9A, B**) [2], [14]. We also assessed whether there was collagen overmodification by steady-state analysis. The electrophoretic migration of type I collagen chains synthesized by wild-type and *Lepre1^H662A/H662A^* fibroblasts was similar and suggested normal post-translational modification (repeated 3 times) (**figure 9C**). These findings differ from the overmodification observed in collagen isolated from *Crtap^−/−^* fibroblasts [2]. These findings support a conclusion that the catalytically inactive P3H1^H662A^ mutant protein can restore the chaperone activity and collagen assembly function of the P3H1 complex in the ER.

## Discussion

We generated and characterized a novel mouse model (*Lepre1^H662A/H662A^*) that harbors a knock-in mutation in the catalytic domain of P3H1. This mutation inactivated the 3-hydroxylase activity of P3H1 and caused loss of 3-Hyp at Pro986 in the collagen α1(I) chain of bone and a reduction to 9% 3-Hyp at Pro986 in the α1(II) chain of cartilage. Our findings are similar to what has been reported in the null mouse mutants for the components of the prolyl 3-hydroxylation complex [2], [14], [15]. Additionally, our data confirm the findings by Pokidysheva et. al. that the Pro986 site in the α1(I) chain of bone is exclusively hydroxylated by P3H1 [21]. The hydroxylation status of the A3 (Pro707) site of the α2(I) chain is similar between the wild-type and *Lepre1^H662A/H662A^* bone (although the wild-type percentage that we observed is lower than that observed by Pokidysheva et. al. and could be attributable to strain differences) and is in contrast to the reduction observed at this site in the *Lepre1^−/−^* bone [21]. The expression of *Leprel1* (encoding P3H2) was found to be dramatically reduced in the bone of *Lepre1^−/−^* mice, possibly explaining the reduction in hydroxylation observed at the A3 site [21]. Pokidysheva et. al. also noted a slight increase in the expression level of *Leprel2* (encoding P3H3), which could account for the residual hydroxylation observed in the *Lepre1^−/−^* mice [21]. Additionally, since the hydroxylation status of the A3 site isolated from bone of *Lepre1^H662A/H662A^* mice is similar to their wild-type littermates, it supports the notion that another P3H is responsible for hydroxylation at this site. The hydroxylation status of Pro986 in the α2(V) chain differed in our *Lepre1^H662A/H662A^* mice compared to *Crtap^−/−^* mice, i.e. 21% hydroxylation in the *Lepre1^H662A/H662A^* mice and <2% hydroxylation in the *Crtap^−/−^* mice [17].

Expression of *Lepre1* (encoding P3H1) varies in cartilage, being highly expressed in the resting/proliferating chondrocytes and less so in pre-hypertrophic chondrocytes (not shown). With high expression of *Leprel1* (encoding P3H2) in pre-hypertrophic chondrocytes and *Leprel2* (encoding P3H3) in the resting/proliferating chondrocytes (not shown), it is possible that the 9% residual hydroxylation of Pro986 in α1(II) in cartilage could be a result of hydroxylation by P3H3 (or P3H2) analogous to the situation in bone with α2(V). Alternatively, it is possible that the histidine to alanine substitution at residue 662 may not completely inactivate the prolyl 3-hydroxylase function of P3H1 although we think this is less likely given published biochemical analysis of this residue and the lack of hydroxylation in bone [20].

The *Lepre1^H662A/H662A^* mice have reduced trabecular bone, but normal cortical bone and normal extrinsic cortical biomechanics. Although the geometric value, cross-sectional moment of inertia, is increased in the *Lepre1^H662A/H662A^* mice, this finding could be a compensatory mechanism to maintain similar extrinsic properties despite inferior intrinsic properties. Due to physiological higher remodeling rates in trabecular bone, a mild effect is more likely to manifest earlier in trabecular bone than in cortical bone, and this could account for the differential phenotype observed in our mice. The mild effect could also account for our histomorphometry data where we observe no difference in osteoid and bone formation parameters and is in contrast to the lower osteoid parameters and bone formation rate observed in the *Crtap^−/−^* mice [2]. Thus, due to a mild effect, we cannot detect a difference in the osteoid and the dynamic indices of bone formation.

Additionally, cartilage is normal in the *Lepre1^H662A/H662A^* mice suggesting that 3-Hyp modification in type II collagen is not required at least in the resting/proliferative zone. **Table 3** compares the bone phenotypes exhibited by *Crtap^−/−^*, *Ppib* ^−/−^, *Leprel1* ^−/−^ and *Lepre1* ^H662A/H662A^ mice. The pronounced growth defects and disorganized growth plates in the mice lacking a functional P3H1 complex may reflect a particular sensitivity of growth plate chondrocytes to ER stress caused by handling misfolded un-partnered subunits of the complex.

**Table 3:** Table comparing *Lepre1^H662A/H662A^* phenotype to established OI mouse models.

End point	*Crtap^−/−^*	*Ppib^−/−^*	*Lepre1^−/−^*	*Lepre1^H662A/H662A^*
Appearance	Rhizomelia, kyphoscoliosis	No rhizomelia, kyphoscoliosis	Rhizomelia, kyphoscoliosis	WT appearance
Growth Curve	Delayed	Delayed	Delayed	No delay
3-Hyp status at Pro986	Absent	Absent	Absent	Absent
Trabecular bone	Decreased	Decreased	Decreased	Decreased
Cortical bone	Decreased	Decreased	Decreased	Normal
Cortical bone biomechanics	Reduced	Unknown	Reduced	Normal
Collagen diameter	Increased	Increased	Decreased	Slight decrease

In contrast to the established KO mouse models, the *Lepre1^H662A/H662A^* mice have a wild-type appearance, no delay in growth curve, and normal cortical bone. In addition, there is a slight decrease in collagen diameter.

The effect of 3-hydroxyproline on collagen stability is not clear. Studies originally suggested the absence of a triple-helix structure for synthetic peptides containing 3-hydroxyproline at all Xaa positions [22], [23]. Recent studies indicate the presence of one or two 3-hydroxyprolines in the Xaa position does produce a triple helix with a consequent slight increase in stability [3], [24], [25]. These data suggest that although the absence of 3-hydroxyproline may not affect the stability of the triple-helix it may alter protein-protein interactions [24]. Since matrix-cell signaling is important in the development and maintenance of connective tissues, it is plausible that collagen post-translational modifications like prolyl 3-hydroxylation could specify protein-protein interactions between collagen and other ECM components. Furthermore, these interactions may be context-dependent differing in trabecular bone vs. cortical bone vs. cartilage. Candidates include ligand interaction sites mapped to the fibril in proximity to Pro986 and include fibronectin and α1β1/α2β1/α1β11 integrins [26].

Mutations that result in loss of the prolyl 3-hydroxylation complex can result in collagen overmodification [27]. This could also independently affect collagen fibril interaction sites, e.g., with small leucine rich proteoglycans (SLRPs) such as decorin. Such disruption or other signaling effects could explain the disorganization of the hypertrophic zone observed in the *Lepre1^−/−^* mice but not observed in the *Lepre1^H662A/H662A^* mice. Future work investigating the signaling defects present in bone and cartilage will be necessary to understand the chondrodysplasia present in patients carrying mutations in *LEPRE1* or *CRTAP*.

Compared with other tissues, the extracellular matrix of bone is unique in the sense that it is able to mineralize [28]. As the dominant component of bone, it is likely that type I collagen plays an important role in the manner of mineralization of the extracellular matrix. It has been argued that the collagen of bone has evolved special features that equip it to constrain the growth internally of nanocrystal plates of hydroxyapatite [27]. Extrafibrillar non-collagenous proteins limit the amount of extrafibrillar crystal growth [27].

Evolutionarily, prolyl 4-hydroxylation increases the thermal stability of the triple helix through hydrogen bonding [29]. Although loss of prolyl 3-hydroxylation is a feature of recessively inherited OI, the evolutionary function of 3-hydroxyproline is still poorly characterized. One clue to the potential role of 3-hydroxyproline in collagen is through the identification of partially occupied 3-Hyp sites in type I and II collagen; these are D-periodically spaced and suggest the modification is involved in some aspect of collagen fibril assembly [30]. Peptide studies suggest that the 3-Hyp residues have selective affinity for one another [30]. Additionally, the 3-hydroxyl groups are outward pointing from the triple-helix, which provides evidence that short-range hydrogen bonding between collagen triple helices is likely to occur [30]. Although 3-Hyp appears early in collagen evolution, the 3-Hyp at Pro986 of the alpha I chain of type I collagen appeared much later [27], [31]. In fact, the presence of 3-Hyp at this residue coincides with the appearance of *CRTAP* and occurs just before the appearance of vertebrates and bone [32]. Taken together, with the potential role of 3-Hyp and the appearance of 3-Hyp at Pro986 just prior to the appearance of vertebrates and bone, we speculate that this unique modification could have functioned to equip collagen molecules for a polymeric architecture that allows organized hydroxyapatite nanocrystal growth within fibrils potentially explaining the lack of phenotype in cartilage vs. bone in our *Lepre1^H662A/H662A^* mice.

In addition to the loss of 3-Hyp at Pro986, we observed an increase in the HP/LP ratio in the bone of the *Lepre1^H662A/H662A^* mice compared to their wild-type littermates. In both dominant and recessive OI, there is an increase in the HP/LP ratio, supporting alterations in collagen crosslinking [33]. The increase in HP/LP ratio suggests a disturbance of the fibrillar architecture in bone and could result in the disorientation of nanocrystal plates of hydroxyapatite, but does not signify overmodification of the collagen. Since we observe no difference in collagen migration from the steady-state collagen analysis, we conclude that there is no gross overmodification in the collagen isolated from the *Lepre1^H662A/H662A^* mice. Additionally, we observe no difference in collagen secretion, providing indirect evidence that the P3H1 complex is able to form and bind to collagen, in contrast to the *Lepre1^−/−^* mice which have a delay in collagen secretion due to failure of complex formation [14].

Future work investigating the contribution of signaling vs. collagen cross-linking defects present in bone and cartilage will be necessary to understand the generalized connective tissue phenotype present in patients carrying mutations in *LEPRE1* or *CRTAP*.

## Materials and Methods

### Ethics statement

All research involving animals was conducted according to the relevant national and international guidelines. Veterinarians supervised animal care according to standard conditions of Baylor College of Medicine Center for Comparative Medicine, a program fully accredited by the Association for Assessment and Accreditation of Laboratory Animal Care International. All mouse work was approved by the Center for Comparative Medicine in conjunction with the Institutional Animal Care and Use Committee.

### Evolutionary Trace

The Evolutionary Trace (ET) is an approach to identify molecular determinants of protein function and to target mutational analysis and protein engineering to the most relevant amino acids of a protein [34]–[38]. To help identify functionally important sites, ET builds a phylogenetic tree from a multiple sequence alignment and then scans the multiple sequence alignment for residue variations that correlate with major evolutionary divergences in the tree (Full residue invariance, or “conservation”, is a special case). The amino acids are ranked according to a score,
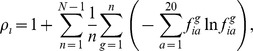
where *f_ia_^g^* is the frequency of the appearance at residue position *i* of the amino acid of type a within the group *g* of subalignments that appear at “distance” *n* from the tree root. 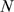 is the number of sequences in the alignment.

In practice, the top-ranked ET residues cluster together spatially in the structure, a property that is used to assess the statistical significance of predictions.

### Fibroblast cultures

Fibroblast cultures were grown in DMEM supplemented with 4 mM L-glutamine, 4500 mg/L glucose (HyClone), 10% FBS, 100units/mL penicillin, and 100 µg/mL streptomycin.

### Lentivirus production and concentration

Viral vector and packaging plasmids were transfected into 293T cells to produce lentivirus, which was concentrated, and transduced into fibroblasts as previously described [39].

### Harvest of cells for protein analysis

After fibroblasts were cultured to confluence in a 60 mm^2^ dish, cells were washed in 1× PBS. Subsequently, cells were scraped in 1 ml 1× PBS and transferred to a 1.5 ml Eppendorf tube. Cells were spun down at 14000 RPM for 5 minutes at room temperature. Samples were resuspended with 50 µl of 5%B-ME and Lamellae loading buffer (Biorad) prior to the separation of proteins on a polyacrylamide gel.

### Immunodetection by immunoblot

10 µg of protein was loaded into each well, run and transferred to a PVDF membrane using a standard wet transfer process. After blocking using 5% milk or 5% BSA, proteins were detected using the following antibodies directed against P3H1, CRTAP, and γ-Tubulin (sigma) diluted in appropriate buffer. The blot was washed 3 times, 10 minutes each with 1× TBS with 0.05% Tween20 and then incubated with the corresponding secondary antibody and washed again. Proteins were visualized using the Luminato Crescendo HRP substrate (Millipore) by incubating blot with the substrate for 2 minutes before exposing the blot to film and feeding it through the developer.

### Immunofluorescence

Fibroblasts were split into glass LAB-TEK 4-well chamber slides (Nunc), and 24 hours later were fixed with 4% paraformaldehyde, treated with 0.1% Triton X-100, blocked in 10% donkey serum and 1% BSA, and then sequentially incubated with 1∶250 dilution of CRTAP antisera or P3H1, 1∶500 donkey anti-rabbit secondary antibody conjugated to Alexa Flour 594 (Invitrogen), and mounted with Prolong Gold anti-fade reagent with DAPI (Invitrogen). The slides were visualized using a Zeiss fluorescence microscope.

### Recombineering

Recombineering was utilized to generate the P3H1 knock-in mouse model using the method described by Pentao Liu [40]. Briefly, retrieval vector and targeting constructs were designed and cloned together in order to retrieve the section of *Lepre1* for gene targeting and for introducing the alanine substitution into the gene locus. The linearized DNA construct was electroporated into AB2.2 embryonic stem (ES) cells and screened for positive recombination events by both Southern blot and sequencing. Germ-line transmission of the mutant allele into C57BL6 was obtained.

### Animal tissue collection and processing

*Lepre1^H662A/H662A^* mice and wild-type littermates were sacrificed at 3 months of age. Spine, femurs and skin were dissected, fixed, paraffin embedded, and sectioned according to standard methods as previously described [2]. The *Lepre1^H662A/H662A^* mouse colony was maintained in a mixed 129Sv/ev-C57Black/6J genetic background and housed in the Baylor College of Medicine Animal Vivarium.

### Radiographs, bone histology/histomorphometry, and tissue staining

Standard protocols were followed for the following stains: Hematoxylin and Eosin. Immunofluorescence on mouse tissues was done as previously described [41]. Briefly, the paraffin sections were xylene treated, rehydrated, and heated for 20 minutes in a steamer for antigen retrieval. Subsequently they were incubated in blocking solution (3% normal Donkey serum, 0.1% BSA, 0.1% Triton X-100 in PBS), 1∶100 dilution of COLX antisera, 1∶600 donkey ant-rabbit secondary antibody conjugated to Alexa flour 594 (Invitrogen), and finally mounted with Prolong Gold anti-fade reagent with DAPI (Invitrogen). At the end of each described procedure, images were captured using a Zeiss Axioplan 2 microscope. Radiographs were obtained by Kubtec XPERT80 (Kubtec X-ray, Milford, CT). Routine histologic analysis of paraffin-embedded long bone and growth plates was done as per standard protocols. Histomorphometric analysis of static and dynamic parameters (using 25 mg/kg calcein injection) of bone resorption, formation, and volume was carried out according to standard procedure in 12 week-old (N = 6 each sex and genotype) [42] and analyzed using the Bioquant OsteoMetrics software system (BIOQUANT Image Analysis Corporation, Nashville, TN).

### Micro-CT analysis

Spine and femur samples were placed into a 16 mm tube filled with 70% ethanol and scanned at 16 micron resolution using a ScanCo uct40 scanner (N = 10 each sex and genotype). Trabecular and cortical analysis was performed using the ScanCo software. The trabecular region of the L4 vertebrae was manually selected (contoured) every 5 slices, and then the remaining slices were morphed to enclose the region of interest, for a total of 100 slices. The region was thresholded at 210 with a gauss setting of 0. Cortical analysis was performed on 50 slices of the femoral midshaft, using the same thresholding.

### Three-point bending analysis

Femurs were collected (N = 7, both genotypes) during tissue harvest, wrapped in gauze soaked in 1× PBS and stored at −20°C until ready for analysis. All testing was performed in a 3-point bending apparatus (Instron 5848) with a span of 7 mm as previously described [14], [43]. The posterior surface of the femur was placed on the lower supports and centered between the two supports. The displacement rate used for the analysis was 0.3425 mm/sec.

Stress was calculated using the following formula:
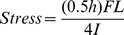
where F is the load applied on the femur in N, L is the span length in mm, h is the specimen diameter in mm, and I is the cross-sectional moment of inertia in mm^4^. L was set at 7 mm for the three point bending jig. Both h and I were obtained by analyzing a midshaft micro-CT image using the slice geometry program in BoneJ, a plugin for bone image analysis in ImageJ. The program identified the bone in the grayscale micro-CT image by pixel brightness and then calculated its cross-sectional parameters once an accurate scale was assigned.

Strain was calculated using the following formula:
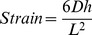
where D is the actuator displacement in mm, h is the diameter in mm and L is the span length in mm.

To determine the Yield Point, a region was identified after the preload and before the maximum load on the Load-Displacement curve. This region was separated into 2 segments from which the fitted line of the segment with greatest slope was taken. Next, a 0.00876 mm offset was implemented on the line. The point of intersection between the offset line and the Load-Displacement curve was the Offset Yield Point. This yield point corresponded to a 0.2% offset strain, which is commonly chosen in the literature. The elastic region was identified as the region from the completion of the preload to the Yield Point. Post-Yield region was identified as the region from the Yield Point until the Failure Point, where the load dropped to zero. Elastic Displacement was the displacement during which specimen remained in elastic region. Post-Yield Displacement was the displacement during which specimen remained in the Post-Yield region. Total Displacement was calculated as the sum of Elastic Displacement and Post-Yield Displacement. Using a trapezoidal numerical integration method, Elastic Energy was calculated as the area under the elastic region of the Load-Displacement curve. Similarly, Post-Yield Energy was calculated as the area under the post-yield region of the Load-Displacement curve. Energy to Failure was the sum of Elastic Energy and Post-Yield Energy. Maximum Load was determined by finding the highest load value recorded by BLUEHILL, before the specimen failed. To calculate Stiffness, Least Square fit method was applied to the steepest segment of the elastic region of the Load-Displacement curve. Stiffness was the slope of least square fit line.

Ultimate Strength was determined by finding the highest Stress value before the specimen failed. Using a trapezoidal numerical integration method, Elastic Toughness was calculated as the area under the elastic region of the Stress-Strain curve. Similarly, Post-Yield Toughness was calculated as the area under the post-yield region of the Stress-Strain curve. Toughness to Failure was the sum of Elastic Toughness and Post-Yield Toughness. To calculate Elastic Modulus, Least Square fit method was applied to the steepest segment of the elastic region of the Stress-Strain curve. Elastic Modulus was the slope of least square fit line.

### Electron microscopy

Freshly dissected tissues were fixed in 1.5% glutaraldehyde/1.5% paraformaldehyde with 0.05% tannic acid in 0.1 M Cacodylate buffer, pH 7.4 for 60 minutes on ice, rinsed in 0.1 M cacocylate overnight, then postfixed for 60 minutes in cacodylate buffered 1% OsO4, rinsed, then dehydrated in a graded ethanol series from 30–100%. The samples were washed in propylene oxide and embedded in Spurrs epoxy. Ultrathin sections were stained in Uranyl Acetate followed by Reynolds lead citrate and examined using a FEI Tecnai G2 TEM. Transmission electron microscopy was performed on skin of wild- type and *Lepre1^H662A/H662A^* mice (N = 3). The fibril diameter of ten fibrils in each of fifteen different areas per mouse was measured (N = 150 total measurements).

### Collagen cross-link and mass spectral analyses

Pyridinoline cross-links (HP and LP) were quantified by HPLC after hydrolyzing demineralized bone in 6N HCl as described [44].

Types I and V collagens were prepared from minced bone decalcified at 4°C in 0.1M HCl overnight. Type I α-chains were extracted by heat denaturation (90°C) in SDS-PAGE sample buffer. Type V collagen was solubilized by pepsin in 3% acetic acid and selectively precipitated by 1.8 m NaCl [45]. Type II collagen was solubilized by CNBr digestion of rib cartilage in 70% formic acid [46]. Collagen α-chains and CNBr-peptides were resolved respectively on 6% and 12.5% SDS-PAGE gels [47].

Demineralized bone matrix was digested with bacterial collagenase as described [48]. Collagenase-generated peptides were separated by reversed-phase HPLC (C8, Brownlee Aquapore RP-300, 4.6 mm×25 cm) with a linear gradient of acetonitrile∶n-propanol (3∶1 v/v) in aqueous 0.1% (v/v) trifluoroacetic acid [49]. Individual fractions were analyzed by LC-MS.

Collagen α-chains or CB peptide bands were cut from SDS-PAGE gels and digested with trypsin in-gel [50]. Peptides were analyzed by electrospray LC/MS using an LCQ Deca XP ion-trap mass spectrometer (ThermoFinnigan) equipped with in-line liquid chromatography using a C8 capillary column (300 um×150 mm; Grace Vydac 208MS5.315) eluted at 4.5 ul min. The LC mobile phase consisted of buffer A (0.1% formic acid in MilliQ water) and buffer B (0.1% formic acid in 3∶1 acetonitrile∶n-propanol v/v). An electrospray ionization source (ESI) introduced the LC sample stream into the mass spectrometer with a spray voltage of 3 kV. Sequest search software (ThermoFinnigan) was used for peptide identification using the NCBI protein database. Large collagenous peptides not found by Sequest had to be identified manually by calculating the possible ms/ms ions and matching these to the actual ms/ms. Hydroxyproline and hydroxylysine calculations were done manually by scrolling or averaging the full scan over several minutes so that all of the post-translational variations of a given peptide appeared together in the full scan.

### Fibroblast isolation from mice

Mice were euthanized using isoflurane, the fur was scraped off the back of the mouse and a small section of skin was harvested. The skin was washed with 1× PBS and placed dermis down onto a well of a 6-well plate. 2 ml of DMEM complete was added to the well and the skin was cut into small pieces using a scalpel to allow fibroblasts to migrate from the skin.

### Collagen secretion from fibroblasts

We analyzed procollagen secretion by pulse-chase assay as previously described [51], [52]. Briefly, we plated 2.5×10^5^ cells onto a 35 mm dish and let them grow overnight. The next evening, the medium was changed to DMEM complete with 50 µM ascorbate to induce collagen synthesis. After washing the cells 3 times with 1×PBS to remove FBS from the cells, the medium was replaced with serum free DMEM containing 50 µM ascorbate and 140 µCi of L-[2,3,4,5-^3^H] proline. Cells were labeled for 1 hour and then chased with fresh medium containing unlabeled proline. Both the cell layer and medium were harvested in 1× PBS containing 1× inhibitor at 20-minute intervals and the procollagen was precipitated with collagen carrier (Sigma) and absolute ethanol. Samples were electrophoresed on a 5% acrylamide gel containing urea under reducing conditions, dried, and imaged by exposing film to gel for 24 hours at −80°C and processed using a developer. Procollagen secretion over time was measured by comparing the amount of labeled procollagen present in the cell layer and medium at each time point. The pulse-chase assay was repeated three times to confirm the results.

### Collagen steady-state analysis

We analyzed collagen modification by collagen steady-state analysis as previously described [51], [52]. Briefly, we plated 2.5×10^5^ cells onto a 35 mm dish and let them grow overnight. The medium was changed to DMEM complete with 50 µM ascorbate to induce collagen synthesis. After 4 hours, the cells were washed 3 times with 1×PBS and the medium was replaced with serum free DMEM containing 50 µM ascorbate and 140 µCi of L-[2,3,4,5-^3^H] proline. Cells were labeled overnight. Both the cell layer and medium were harvested in 1× PBS containing 1× inhibitor and the procollagen was precipitated with collagen carrier (Sigma) and absolute ethanol. Collagens were obtained by overnight pepsin digestion (50 ug/ml) of procollagen samples. Samples were electrophoresed on a 5% acrylamide gel containing urea under reducing conditions for collagen samples, dried, and imaged by exposing film to gel for 24 hours at −80°C and processed using a developer. This assay was repeated three times to confirm the results.

### Statistical analyses

Data are expressed as mean values ± standard deviation (SD). Statistical significance was computed using the Student's t test. A P value <0.05 was considered statistically significant.

## Supporting Information

Figure S1 Generation of *Lepre1^H662A^* mutant allele. A recombineering strategy was utilized to incorporate the H662A mutation into the *Lepre1* gene locus. Positive recombination events were analyzed by Southern blot using XmnI to differentiate the genomic locus allele from the targeted (*Lepre1^H662A^*) allele.
(TIFF)

Figure S2 *Lepre1^H662A/H662A^* mice have a normal tibial hypertrophic zone. Since the *Lepre1^−/−^* animals showed disorganization of the hypertrophic zone, we assessed the hypertrophic zone (P1) of *Lepre1^H662A/H662A^* mice by H&E staining (A) and by specifically marking the hypertrophic zone using an antibody directed against type×collagen (B). The hypertrophic zone of the tibia of the *Lepre1^H662A/H662A^* mice are indistinguishable from their wild-type littermates (B) and this is confirmed by quantifying the width of the hypertrophic zone in which there is no difference in the width between genotypes (C) (N = 10, both genotypes).
(TIFF)
