# Correction: Differential Effects of Collagen Prolyl 3-Hydroxylation on Skeletal Tissues

**DOI:** 10.1371/journal.pgen.1004473

**Published:** 2014-06-03

**Authors:** The PLOS Genetics Staff

Figure 9 is incorrect. It contains components that are duplicated and do not correspond to what is stated in the figure legend or in the text. Specifically the *Lepre1^H662A/H662A^* gel in panel A is a duplication of the *Lepre1^+/+^* gel, and the *Lepre1^+/+^* lane in panel C is a duplication of the *Lepre1^H662A/H662A^* lane. The authors have provided a corrected version here. This corrected version corresponds to what is stated in the figure legend and in the text.

**Figure 9:**
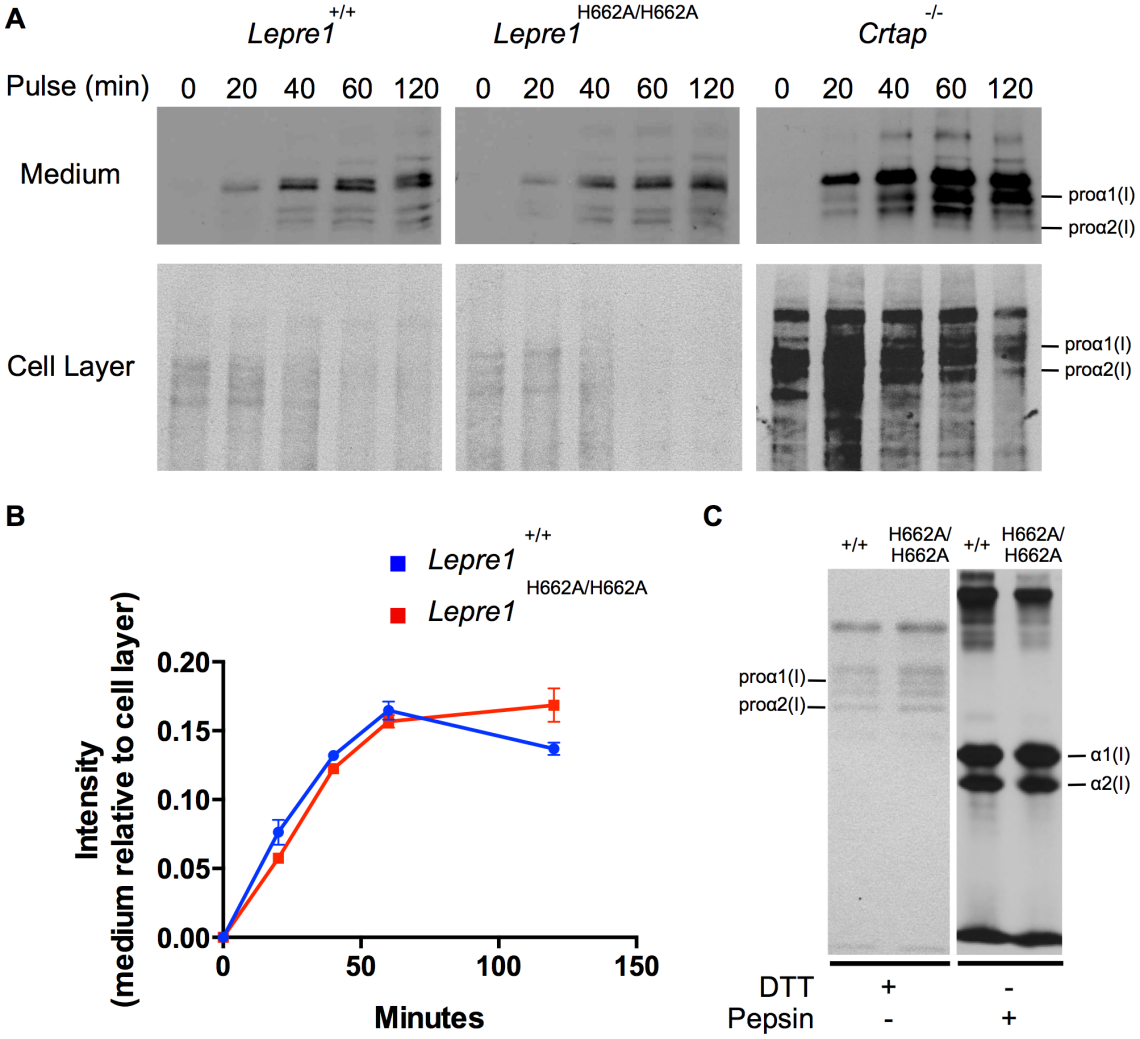
*Lepre1^H662A/H662A^* fibroblast procollagen secretion rate and collagen modification is normal. Analysis of procollagen secretion by the collagen pulse-chase assay suggests that the procollagen secreted from *Lepre1^H662A/H662A^*fibroblasts is similar to *Lepre1^+/+^* fibroblasts (A, B). Additionally, there does not appear to be a decrease in the amount of procollagen secreted from the *Lepre1^H662A/H662A^* fibroblasts in comparison to*Lepre1^+/+^* fibroblasts (A, B). These findings are in contrast to that of the *Crtap^−/−^* fibroblasts, which have an increase in the rate of procollagen secretion (A). Collagen modification was assessed using the collagen steady-state assay. We observed no difference in the migration pattern of procollagen and collagen isolated from *Lepre1^+/+^*(+/+) and *Lepre1^H662A/H662A^* (H662A/H662A) fibroblasts (C). These assays were repeated three times.
