# Supplementary figures and images for: Differential Effects of Collagen Prolyl 3-Hydroxylation on Skeletal Tissues
Source: PLoS Genet. 2014 Jan 23;10(1):e1004121. doi: 10.1371/journal.pgen.1004121 (PMC3900401; doi:10.1371/journal.pgen.1004121)

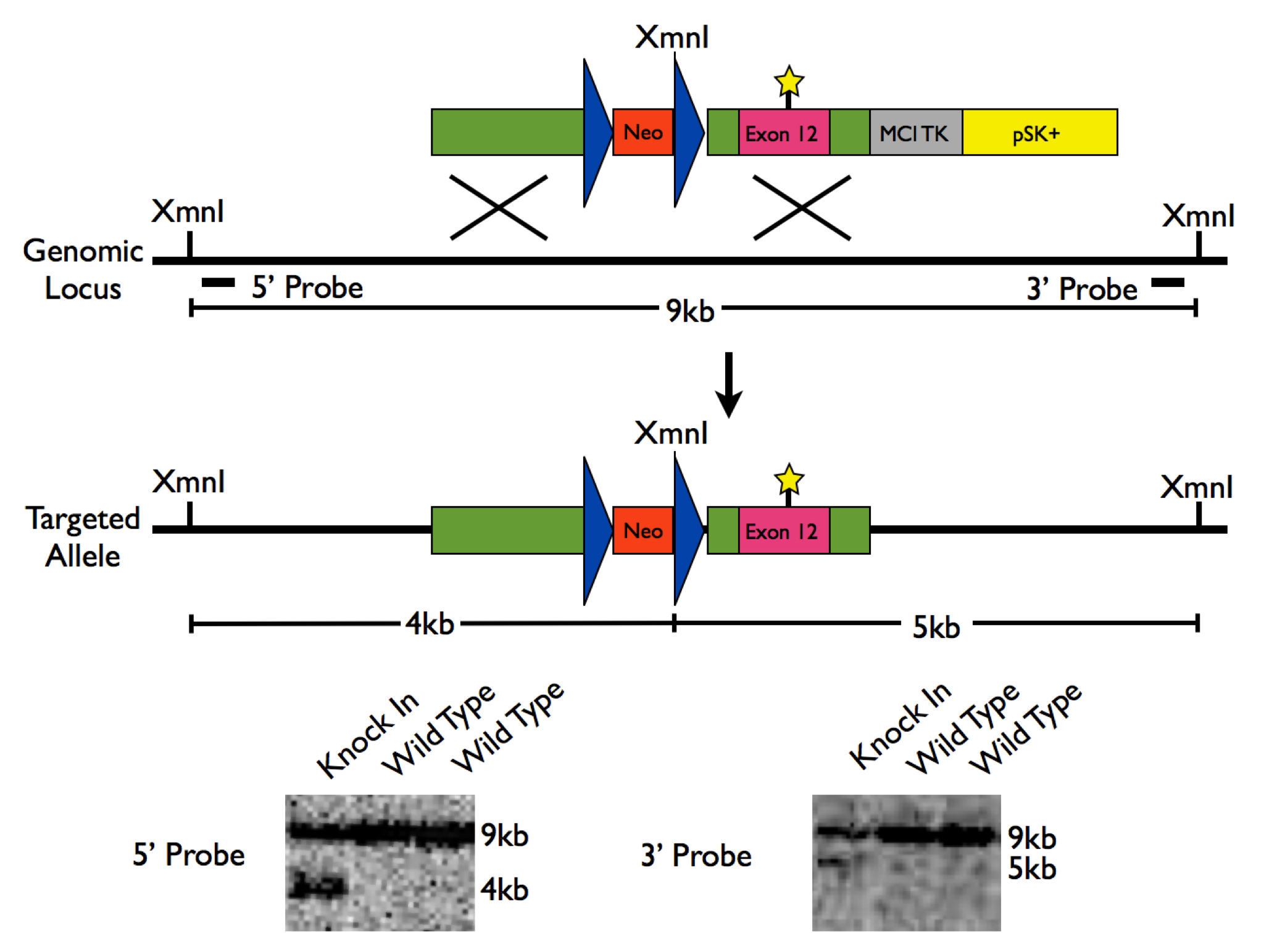

Supplement: Figure S1 — Generation of Lepre1H662A mutant allele. A recombineering strategy was utilized to incorporate the H662A mutation into the Lepre1 gene locus. Positive recombination events were analyzed by Southern blot using XmnI to differentiate the genomic locus allele from the targeted (Lepre1H662A) allele. (TIFF) [file pgen.1004121.s001.tiff]

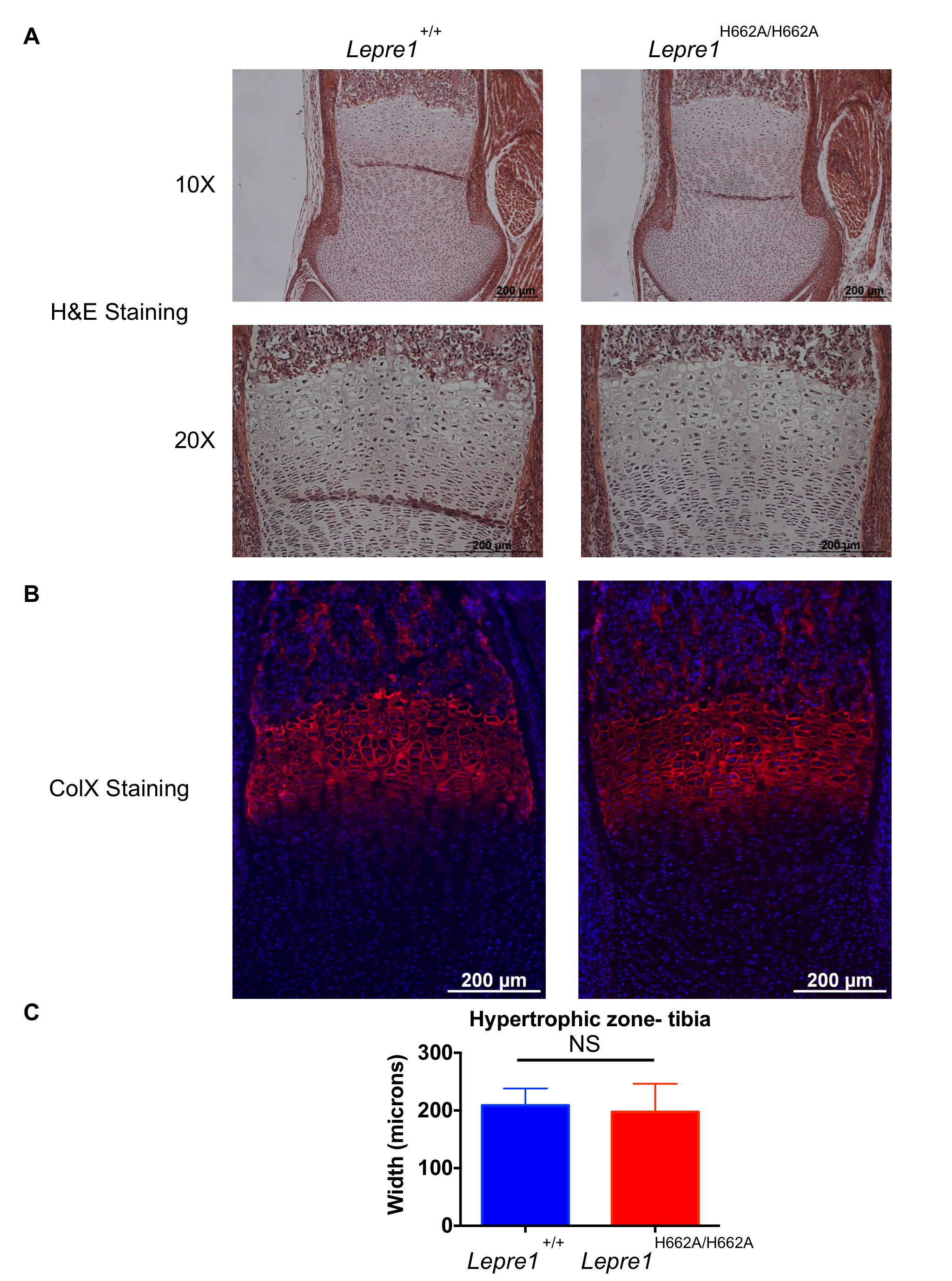

Supplement: Figure S2 — Lepre1H662A/H662A mice have a normal tibial hypertrophic zone. Since the Lepre1−/− animals showed disorganization of the hypertrophic zone, we assessed the hypertrophic zone (P1) of Lepre1H662A/H662A mice by H&E staining (A) and by specifically marking the hypertrophic zone using an antibody directed against type×collagen (B). The hypertrophic zone of the tibia of the Lepre1H662A/H662A mice are indistinguishable from their wild-type littermates (B) and this is confirmed by quantifying the width of the hypertrophic zone in which there is no difference in the width between genotypes (C) (N = 10, both genotypes). (TIFF) [file pgen.1004121.s002.tiff]
